# Diabetes Mellitus—A Risk Factor for Unfavourable Outcome in COVID-19 Patients—The Experience of an Infectious Diseases Regional Hospital

**DOI:** 10.3390/healthcare9070788

**Published:** 2021-06-23

**Authors:** Egidia Miftode, Larisa Miftode, Ioana Coman, Cristian Prepeliuc, Maria Obreja, Oana Stămăteanu, Tudorița Gabriela Părângă, Daniela Leca, Claudia Elena Pleşca

**Affiliations:** 1 “Grigore T. Popa”, University of Medicine and Pharmacy Iasi, Discipline of Infectious Diseases, 700116 Iasi, Romania; egidia.miftode@umfiasi.ro (E.M.); maria.n.obreja@d.umfiasi.ro (M.O.); lidia-oana.stamateanu@umfiasi.ro (O.S.); daniela.leca@umfiasi.ro (D.L.); claudia-elena.badarau@umfiasi.ro (C.E.P.); 2 “Saint Parascheva”, Infectious Diseases Clinical Universitary Hospital Iasi, 700116 Iasi, Romania; ioana.coman17@gmail.com (I.C.); cristiprep94@gmail.com (C.P.); tudorita_paranga@email.umfiasi.ro (T.G.P.)

**Keywords:** cytokine storm, diabetes mellitus, COVID-19

## Abstract

Early research into the implications concerning the evolution of the infection caused by the new coronavirus in people with glucose metabolism dysfunction, in this case diabetics, shows that severe forms of the disease predominate in this risk category. Moreover, it seems that even in patients with normal glycaemic status, COVID-19 may predispose to the development of hyperglycaemia which modulates immune mechanisms and inflammatory responses, with direct effects on morbidity and mortality. Thus, taking into account these scientific data, as well as the increased frequency of diabetes in the general population, we aimed to assess the risk of an unfavourable outcome of diabetic patients, which is in a strong connection with the presence and severity of pulmonary disease such as interstitial pneumonia/bronchopneumonia, as well as the effectiveness of Tocilizumab administration. The results of our study indicate a three-fold higher risk of death in patients with diabetes and COVID-19 (RR = 3.03; IC95%: 2.37–3.86; *p* = 0.001),compared to nondiabetic patients, and the risk of developing severe forms of acute respiratory failure was 1.5 times higher in the first studied category. In conclusion, we can say that the diabetic diagnosed with SARS-CoV-2 infection is more predisposed to immunological and organic dysfunctions that may ultimately result in death, and treatment with monoclonal anti-IL-6 antibodies was more effective in diabetic patients than non-diabetics (*p* < 0.05). The effectiveness of Tocilizumab was significant in both studied groups, but diabetic patients responded better to this therapy compared to non-diabetes-mellitus (DM) ones (76.7% vs. 35% *p* = 0.001).

## 1. Introduction

Diabetic patients with COVID-19 have an increased risk of severe pneumonia and a more pronounced pro-inflammatory and pro-thrombotic status compared to non-diabetics infected with SARS-CoV-2. These COVID-19 patients with uncontrolled hyperglycaemia have a particularly high mortality rate. Diabetes mellitus is per se a risk factor for the progression to severe forms and even death. In addition, it seems that, even in patients with normal glycaemia, COVID-19 can predispose to hyperglycaemia which can also modulate the immune mechanisms and inflammatory responses, with direct effects on mortality and morbidity [1].

The number of deaths among COVID-positive patients over a period of approximately 11 months, as well as the associated acute or chronic pathologies that contributed to their unfavourable outcome, were variable. Thus, in most cases, it was flu and lower respiratory tract infections, followed by comorbidities such as hypertension, diabetes mellitus, chronic renal failure, heart rhythm disorders, heart failure, neoplastic diseases, and obesity [2,3,4].

The mechanisms accounting for the triggering of hyper-inflammatory phenomena in COVID-19 are complex and incompletely elucidated. According to the latest research on this subject, it appears that infection of epithelial cells in the airways and virus replication in these tissues cause high levels of apoptosis/pyroptosis, triggering important inflammatory responses marked by the activation of pro-inflammatory cytokines/chemokines. As a result, macrophages and monocytes are recruited at the site of infection, activation of T and B lymphocytes occurs, with the cellular response leading to immune control of infection in most cases. However, in some individuals, immune disorder induces an insufficient response of interferon type I (IFN), aberrant secretion of pro-inflammatory cytokines by alveolar macrophages, and subsequent dysfunction of CD4 and CD8 T cells [5].

Given the chronic pro-inflammatory status found in diabetic patients, it is understandable that even the presence of typical complications of diabetes mellitus (chronic renal failure, chronic heart dysfunction, and stroke) constitutes an addition to the mortality generated by COVID-19 [6,7,8]. The mechanisms by which glycaemic disorders favour the occurrence of the inflammatory cascade are multiple and complex. Natural Killer (NK) cells and gamma interferon are known to play a decisive role in increasing vascular and/or interstitial permeability for pro-inflammatory cytokines. As a harmful complement to these reactions, SARS-CoV-2 infection constantly generates the production of oxygen free radicals (OFR), the immediate effect of which is to induce pulmonary fibrosis; acute respiratory distress syndrome; and respiratory, cardiovascular, and renal collapse [9].

## 2. Material and Methods

Our retrospective case-control study included 3985 COVID-19 confirmed patients admitted to “Saint Parascheva” Infectious Diseases Clinical Hospital in Iasi between March and November 2020. The inclusion criteria were age over 18 years and the diagnosis of SARS-CoV-2 infection confirmed by Real Time Polymerase Chain Reaction (RT-PCR). The patients under 18 years old and with a RT-PCR negative for SARS-CoV-2 were not eligible for our study. All patients were evaluated in terms of age, gender, lung disease, admission to the intensive care unit, need for Tocilizumab as a result of the installation of cytokine storm and progression to death depending on the association of diabetes mellitus (DM). Increased values of inflammatory markers (C reactive protein-CRP, Fibrinogen), Interleukin 6 (IL-6),lactate dehydrogenase (LDH), d-dimers and lymphopenia, and thrombocytopenia, as well as the presence of fever, dyspnoea with orthopnoea, and decreasing peripheral oxygen saturation measured by pulse oximetry were considered defining elements of inflammatory complication specific for SARS-CoV-2 infection (cytokine storm). All patients had at least a chest X-ray in order to establish the form and severity of lung injuries (unilateral/bilateral interstitial infiltrates suggestive of interstitial pneumonia or confluent nodular opacities suggestive of bronchopneumonia), if not a thoracic computer-tomography (CT-scan) to put in evidence-specific aspects of COVID-19 pneumonia (ground glass opacities with peripheral and subpleural distribution and involving the lower lobes, predominantly). A severe form of COVID-19 was defined by the presence of severe pneumonia (more than 50% of both lungs affected), decreasing oxygen saturation under 90% in the atmospheric air. The patients’ personal and medical data were anonymized before usage. We used the STROBE guidelines to guarantee the quality of the study [10]. This study was approved by the ethics committee of the hospital (approval number 22/31st of March 2021). Each patient also signed an informed consent with reference to the methods of investigation, applied therapy, and access to personal data.

Statistical data processing was carried out using the SPSS 18.0 programme at a 95% threshold for statistical significance. The χ^2^ test was used as a qualitative nonparametric test to compare frequency distributions. A number of derived indicators described by ANOVA tests were used such as: mean, median, minimum/maximum values, standard deviation, standard error, and variation coefficient. We have also used linear regression analysis to assess correlations between SARS-CoV-2 infection and mortality predictors.

## 3. Results

The total number of patients diagnosed with COVID-19 and hospitalized at the Regional Infectious Diseases Hospital in Iasi between March 2020 and November 2020 was 3985,resulting in 250 deaths (6.4%). The total number of patients with diabetes mellitus was 634 (16.3%),resulting in 91 deaths (14.5%). Of the total number of deaths during this period (*n* = 250), 36.4% were patients with type 2 diabetes mellitus. The lowest number of deaths was noted in March (4%), with an increasing trend in the following months, reaching a peak in October (22.6%) (Table 1).

The estimated risk of death in diabetic patients with SARS-CoV-2infection was three times higher than in patients who were not associated with this metabolic disorder (RR = 3.03; IC95%: 2.37–3.86; *p* = 0.001) (Table 2). The risk of progression to severe forms of SARS-CoV-2 infection was also 1.5 times higher for those with diabetes mellitus (RR = 1.51; IC95%: 1.32–1.73; *p* = 0.001) (Table 2).

**Figure 1:**
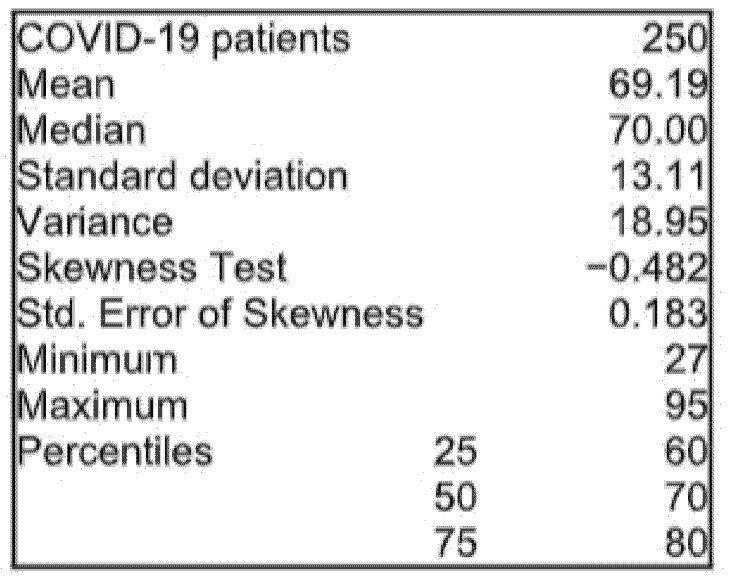
Statistical indicators for age groups.

The mean age of deceased patients with COVID-19 was 69.19 years ± 13.11 (Figure 1). The median age of these patients was close to the mean value. Moreover, the age range was homogeneous (Skewness test > −2).

The deaths’ frequency peak was recorded in the age group 70–79 years (28.2%), but high frequencies should also be noted in the age group 60–69 years (23.2%) and 80–89 years (22%) (Table 2).

The frequency of deaths was higher in males (70%), but in both sexes there was a higher mortality among elderly patients (69.9%, *p* = 0.037) (Table 3).

Some of our COVID-19 diabetic patients presented other comorbidities also, as can be observed in Table 4. Among these chronic diseases, the most representative were High Arterial Blood Pressure, Obesity, Chronic Kidney Disease, Neoplasia, Autoimmune diseases, and Liver Cirrhosis. Seventy-five patients (11.8%) did not have any other comorbidity, except type 2 diabetes mellitus.

A percentage of 36.2% of COVID-19-deceased patients had diabetes mellitus, the most affected being the males (57.8%; *p* = 0.759) and the age group over 65 years (71.9%; *p* = 0.447), as well as those hospitalized in the intensive care unit of our hospital (71.9%; *p* = 0.879) (Table 5).

Regarding the presence of lung involvement such as interstitial pneumonia or bronchopneumonia, the association with diabetes mellitus did not induce a higher estimated risk in patients with COVID-19, as shown in Table 6.

The percentage of diabetic patients who developed bronchopneumonia was 3.2%, identical to that of nondiabetics.The severe form of COVID-19 was present in 30.4% cases of diabetic patients and 20.2% of those without DM (Table 6). We also mention that the whole group of 870 patients who developed severe forms of COVID-19 received corticotherapy with Dexamethasone for at least 10 days. A percentage of 8.4% of COVID-19 patients developed interstitial pneumonia during the disease course, and 8.5% of them had diabetes. Of note, the number of deaths in COVID-19 patients was relatively constant during the first five months of the studied period, the phenomenon being more frequently recorded in the intensive care unit, but in August-October the percentage of deaths in those with diabetes at the time of SARS-CoV-2 infection diagnosis increased significantly. Sixty-eight percent of diabetic patients with COVID-19 had increased values of IL-6 (40.7%; *p* =0.001), and 38.7% of non-DM patients presented these modifications (Table 7).

**Figure 2:**
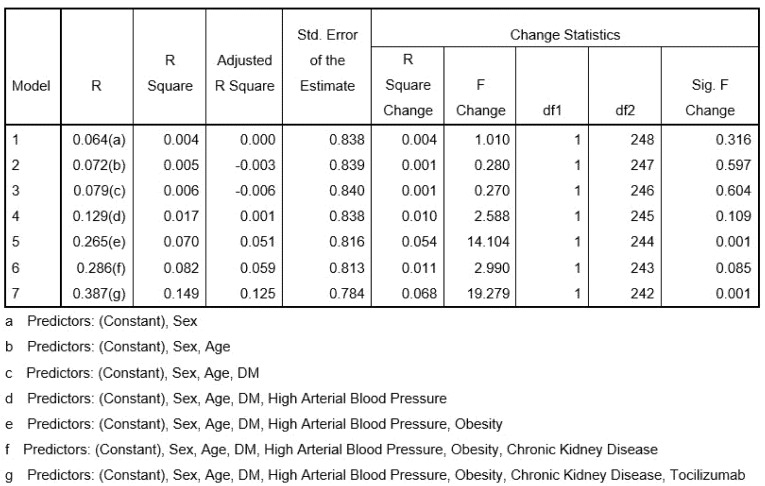
Mortality predictors in SARS-CoV-2 infection.

Treatment with Tocilizumab was initiated in 349 patients (9%) with severe evolution, of which 103 (29.5%) with diabetes mellitus selected on the basis of severity criteria: sudden desaturation, increased CRP, ferritin, D-dimers, LDH, and interleukin 6 values (Figure 2). Most patients received a single dose of Tocilizumab, the second dose being administered only when a patient’s status fails to improve. The third dose was exceptionally administered.

Regarding the need for Tocilizumab in patients diagnosed with COVID-19, there were notable differences between the categories of those with diabetes mellitus vs. non-diabetics. Thus, 16.2% (103 out of 634) of patients with DM needed an IL-6 antagonist as compared to those without DM, 7.3% of whom (246 out of 3351) required Tocilizumab (Chi 2 = 52.89; *p* = 0.001) (Table 6). Moreover, the favourable outcome in patients receiving Tocilizumab was noted in a significantly higher proportion in diabetics than in non-diabetics (76.7% vs. 35%; *p* = 0.001) (Table 8).

As for the hypoglycaemic therapy given to diabetic patients with COVID-19, all patients received insulin during hospitalization, even those previously treated with oral antidiabetics. In cases where glycaemic values had normalized, the option of resuming oral therapy was considered, in accordance with the recommendations of the diabetes specialist. Regression analysis showed that sex, age, diabetes mellitus, high arterial blood pressure, and obesity are good predictors for the unfavourable outcome in patients with SARS-CoV-2 infection (Figure 2).

## 4. Discussion

As the latest research on this topic indicates, however, sepsis is the most common complication in patients with SARS-CoV-2 infection as a result of direct viral involvement. The first phase of evolution associates more frequently with the pro-inflammatory reaction of immune system activation, and the second consists of a process of cell-mediated immunosuppression. Activation of lymphocytes aims to achieve viral clearance by means of cytokines and specific antibodies. According to researchers, interleukin 6 (IL-6) plays a leading role in the cytokine storm, and its significantly elevated levels may correlate with a high mortality rate in affected individuals [11].

On the other hand, comorbidities such as diabetes have been reported as major risk factors for the adverse development in the two types of coronavirus infections that preceded the COVID-19 pandemic (SARS and MERS—Middle East Respiratory Syndrome). Information on the immunological mechanisms triggered by the new coronavirus in diabetic patients is still incomplete and under analysis. It appears that this population category is more likely to develop hyper-inflammatory responses and much more severe forms of disease, and the likelihood of admission to intensive care wards is also increased [12]. The reported data show that the incidence of diabetes among patients diagnosed with SARS-CoV-2 infection varies between 7.4% and 20% [13,14]. Still, the majority of our patients with type 2 diabetes mellitus (89.7%) associated with various other comorbidities, which could also increase the mortality and morbidity rate.

In the observational study that we conducted between March and November 2020, we noted an increased percentage (16.3%) of diabetic patients, 36.4% of whom died.

SARS-CoV-2 infection may develop in three successive stages: stage I (early phase of infection), stage II (pulmonary phase), and stage III (hyper-inflammatory phase) [15].

One of the main triggering factors of adult acute respiratory distress syndrome (ARDS), responsible for increasing the morbidity and mortality rate among patients with SARS-CoV-2 infection, is the cytokine storm. This represents an uncontrolled, often deadly systemic inflammatory response resulting from the release of large amounts of pro-inflammatory cytokines (IFN-α, IL-1b, IL-6, IL-12, IL-18, TNF-α, etc.) and chemokines by immune effector cells in SARS-CoV-2 infection [16,17,18,19].

In the pulmonary phase, some patients develop shortness of breath and abnormal thoracic radiological images and increased transaminases, and in the advanced, more severe phase, a category of patients develop the so-called “cytokine storm”, the foundation for other life-threatening complications such as acute respiratory distress syndrome, shock, multiple organ dysfunction, and even death. What is particular to this stage is the increase in inflammatory markers (CRP, LDH, IL-6, D-dimers, ferritin, prothrombin, troponin, NT pro BNP), as well as marked lymphopenia [20,21].

Zheng M et al. demonstrated in their study the relationship between the pro-inflammatory status of patients diagnosed with COVID-19 and the glucose metabolism. It seems that diabetic patients diagnosed with SARS-CoV-2 infection had higher values of IL-2, IL-6, IL-10, and interferon gamma than those without diabetes mellitus (NDM) or impaired fasting glucose (IFG). The mechanisms responsible for the cytokine storm are often due to dysregulation in the glucose metabolism. The ICU admission rate for the DM group was also higher than the other studied groups [22].

We have also noticed that persons suffering from diabetes mellitus tend to be more affected during COVID-19 (30.4% vs. 20.2%) by developing more severe forms of disease, but the rates of ICU admission in both studied groups were quite similar (71.9% vs. 70.8%).

It is well known that endogenous glucocorticoids play an essential role in various metabolic and inflammatory processes. In addition to that, the effectiveness of synthetic glucocorticoids has been proven in various immune-related pathologies or viral infections in which the host’s immune response is exaggerated. Although the corticoids administration in patients with SARS-CoV-2infection was initially controversial, the ultimate guidelines included this type of anti-inflammatory therapy as an important step for complication prevention and treatment. Not only in COVID-19, but in other viral infections, Dexamethasone and methylprednisolone were meant to inhibit inflammation through their effects on macrophages [23]. All our patients with and without DM who developed severe forms of COVID-19 and associated acute respiratory failure received not only oxygen therapy, but also dexamethasone. This aspect could be related to glycaemic metabolism disorders, especially in patients with DM and to an unfavourable outcome also.

These findings are consistent with what has been observed in the case of SARS and MERS in the sense that the presence of lymphopenia and the excessive inflammation that defines the host’s immune response plays a major role in the pathogenesis of COVID-19. Therefore, the severity of the disease is due not only to viral infection, but also to the response of the host [17,24].

Regarding the gender distribution of patients with COVID-19 and diabetes, the proportions are relatively equal, and the mean age of diabetics was higher than in patients who did not present this comorbidity. Our study highlights the predominance of the male sex in both groups of patients, with the indication that the average age of over 70 years of diabetic patients was an additional risk factor for any adverse development. In Italy, deaths occurred mainly in the age group 70+ years. Certainly, a great number of them had other comorbidities also—cardiovascular, renal, pulmonary—which led to death [25].

Acheampong, D.O., et al. have studied the aspects referring to males’ predisposition of developing severe forms of SARS-CoV-2 infection and concluded that an exaggerated immune response to viral infections known as the cytokine storm was responsible for their unfavourable outcome. These physio-pathological processes seem to be sustained by androgen hormones which, unlike estrogen, can activate immune cells in an infectious context [26].

The comparative study of the immune response in the two sexes by Takahashi reveals that there are important differences consisting of higher levels of cytokines IL-8 and IL-18 in men, while in women there was a more intense reactivation of T cells [25,27]. In our study, the mortality rate was higher in men, irrespective of age group.

A possible mechanism by which diabetes can increase the risk of infection is a significant viral load due to the effective entry of the virus into the cells in the context of an increased expression of ACE2 in the lungs, kidneys, and heart. High blood glucose levels can increase glucose concentrations in respiratory secretions, and, as a result of this metabolic phenomenon, viral replication in pulmonary epithelial cells is greatly accelerated. Although it remains to be determined whether hyperglycaemia increases SARS-CoV-2 in an in vivo replication, this may partly explain the prolonged recovery of diabetic patients who developed COVID-19 [28]. High glucose levels can also suppress the antiviral response of the host organism.

Thus, the respiratory dysfunction present in people with diabetes, along with the predisposition of SARS-CoV-2 to infect lung tissue cells, may aggravate the pulmonary complications of COVID-19. In addition, the endothelial dysfunction observed in people with diabetes contributes to the onset of the cytokine storm and the occurrence/aggravation of lung damage [29,30].

People with diabetes, regardless of the progression stage of the disease, have a major infectious potential. Diabetes mellitus is considered per se an independent risk factor for the respiratory tract infections’ evolution [31].

Yang et al. studied mortality-related issues among patients with diabetes compared to non-diabetics and tried to establish the importance of hypoglycaemic therapy in improving the prognosis of those in the first category. The mortality rate was two to three times higher in diabetics with SARS-CoV-2 infection. It appears that the mortality predictors in this category of patients were old age and elevated C-reactive protein (CRP) [32]. Loss of autonomic innervation is a plausible explanation for the alteration of the respiratory response to stimuli and for the unfavourable evolution of respiratory infections [33].

A high mortality rate was also recorded in patients with SARS-CoV-2 infection included in our study, a third of whom were diabetics. A total of 71.9% of deaths in this population category were people over 65 years of age. Male patients also experienced a more severe development. Thus, by corroborating the results of international clinical trials with those obtained from the research carried out in our clinic, we can state that there is a risk of death at least double in the category of patients with diabetes with which, in the current epidemiological context, infection with the new coronavirus is associated.

Another important aspect is that of types of medication with a role in regulating glycaemic metabolism in patients with COVID-19. There is controversy in the sense that although insulin therapy is generally preferred during the course of infections in general, some studies have indicated a reserved prognosis in diabetic patients who are COVID-positive. Two possible explanations would be either a negative effect of insulin administration or the peculiarities of patients who received this treatment [34,35].

Our study does not confirm this, as all diabetic patients with severe forms of the disease received insulin therapy during hospitalization, due to the need for adequate blood glucose control, especially in the context of the development of the cytokine storm. Thus, the favourable evolution of 76.7% of all patients with diabetes mellitus may be the result, first of all, of the treatment with Tocilizumab, with an indirect effect on glycaemic metabolism.

Irrespective of the degree of endothelial cell involvement, the initial delay in the gamma interferon response along with the hyper-inflammatory response in diabetic patients may exacerbate the “cytokine storm” and amplify the severity of COVID-19. In particular, endothelial capillaries in basal lamina and alveolar epithelial cells have been described as significantly thicker in people with diabetes than in controls [36,37].

By corroborating these data, it can be stated that patients with diabetes are much more vulnerable to thrombotic events during inflammatory states. Wu L. et al. proposed four risk factors that may increase the risk of severe evolution in patients with diabetes mellitus: susceptibility to hyperglycaemia during corticosteroid treatment, inadequate glucose monitoring, lack of specialist consultation, and inadequate dysregulation of angiotensin receptor blockers. The corticotherapy-related controversy has strong implications not only in patients with apparent contraindications such as diabetics, but also in all types of virus-related pathologies. In clinical practice, it is well known that a short-term, low-dose corticotherapy represents a common option in cases of excessive systemic inflammation and multi-organ dysfunction induced by a viral agent. Critical patients with COVID-19 who develop acute respiratory distress syndrome (ARDS) are part of the anterior-mentioned scenario, in which intravenous glucocorticoids play the role of decreasing lungs’ inflammation and replacing the adrenal function. Despite general recommendations, the administration of corticoids in diabetics with SARS-CoV-2 infection should be carefully argumented because of the potentially induced hyperglycaemia [38].

All our COVID-19 patients with acute respiratory failure received corticotherapy consisting of low doses of intravenous dexamethasone, and, only in cases of rapid disease progression, the treatment with Tocilizumab was taken into consideration. At the same time, the blood glucose levels were monitored and adjusted as the diabetologists recommended, and, in most of the cases, oral antidiabetic drugs were replaced by insulin therapy, considered to be the optimal solution for counteracting the side effects of systemic glucocoricoids. The general mortality rate among diabetic patients with COVID-19 (36.4%) compared to the mortality rate of DM patients who received Tocilizumab (23.3%) brings us new therapeutic perspectives.

It has been demonstrated that carbohydrate metabolism dysfunction in diabetes correlates significantly with the cytokine storm, and the final results, in the absence of adequate therapy, consist of multiple organic failure. Leukocytes activated by exogenous and endogenous stimulus are thought to have the ability to extract glucose independently of the regulating action of insulin. Glycaemic oscillations induce the production of endothelial cytokines and adhesion molecules, which in turn appear to cause uncontrolled extravasation of leukocytes in the alveoli during influenza virus infection, leading to lung damage and impaired respiratory function [39].

Because people with diabetes are at increased risk of a more pronounced inflammatory response, they may be at a higher risk of developing clotting abnormalities. The results of the studies showed that hyperglycaemia led to a pronounced activation of coagulation, and the degranulation of neutrophils was diminished. For these above-mentioned reasons, a good glycaemic profile obtained by oral treatment with antidiabetics, or by appropriate insulin therapy, constitutes a positive prognostic factor in the patient with diabetes mellitus and COVID-19 [40].

In the vast majority of cases hospitalized in the Infectious Diseases Clinic of Iasi, the evaluation and regulation of glycaemic metabolism in patients with marked hyperglycaemia was a therapeutic priority. The objective was achieved through an interdisciplinary collaboration involving the diabetes specialist, the infectious diseases specialist, as well as the intensive care physician.

International studies have shown a more common favourable outcome in patients with uncomplicated and balanced diabetes mellitus, the differentiation being made also in terms of glycosylated haemoglobin values (HaemoglobinA1 C).

As part of the category of immunomodulatory molecules, Tocilizumab has occupied, since the onset of the COVID-19 pandemic, an important role in correcting the hyper-inflammatory response generated by the new coronavirus, especially in the context of the questionable effectiveness of the majority of potentially active antiviral drugs. The mechanism of action of this monoclonal antibody is aimed at blocking interleukin 6 receptors, considered as the key element in the cytokine storm manifested in most patients with severe forms of SARS-CoV-2 infection [41].

In a retrospective study coordinated by Hadjadj et al., the effect of Tocilizumab was evaluated in hyperglycaemic patients with COVID-19 moderate-severe forms. It was noted that IL-6 levels in hyperglycaemic patients were significantly higher than those recorded in patients with normal basal blood sugar levels. Following Tocilizumab administration, IL-6 levels decreased in both groups, but remained higher in the hyperglycaemia group [42]. Our study indicates a high percentage of diabetic patients with increased IL-6 values, compared to non-DM patients (68.1% vs. 38.7%). This could also explain the differences in the mortality rate between the two studied groups.

An extremely important aspect in obtaining the benefits of Tocilizumab treatment in patients with COVID-19, with or in the absence of diabetes mellitus, is the time of administration in relation to the onset of the cytokine storm. Thus, a therapy initiated late, after marked impairment of respiratory function, significantly reduces the chances of survival. Regarding possible adverse reactions of Tocilizumab, most studies indicate low percentages (below 13%). Diabetic people are at greater risk of developing respiratory or systemic fungal infections, bacteremia, fever, cough, and dyspnea in this therapeutic context. On the other hand, the concomitant administration of several types of medication makes it difficult to attribute adverse effects to a single drug [43].

Although there are studies that have not reported an improvement in oxygenation after taking Tocilizumab, the majority of patients hospitalized in our clinic who received immunomodulatory therapy experienced a marked and immediate improvement in clinical parameters, including oxygen saturation. In patients with diabetes mellitus, personalized hypoglycaemic treatment also contributed greatly to the favourable prognosis.

In our study, IL-6 levels were significantly increased in both COVID-positive diabetic and non-diabetic patients with severe forms of disease, as a result of viral pathological implications, but it was interesting to observe clinical and biological improvements after Tocilizumab administration, especially. One of the possible explanations is that of optimizing immunomodulatory therapy through early initiation, from the first signs of the appearance of the cytokine storm. The risk of unfavourable outcome was directly proportional to the degree of delay, with the 23.3% diabetic patients with COVID-19 who died in this category, despite the initiation of IL-6 antagonist treatment.

It is also known that, beyond its effectiveness in reducing the inflammation level, the use of Tocilizumab brings an important benefit in patients with COVID-19 pneumonia, who could develop a refractory hypoxemia [44]. Among the 870 non-DM patients with acute respiratory failure, 349 (40.1%) received Tocilizumab. A number of patients (*n* = 103; 53.3%) from the total of 193 diabetic COVID-19 patients were also treated with the same molecule. This aspect could explain the favourable outcome of this category (76.6% survivors), but the poor response to Tocilizumab in the non-DM group is possibly due to the therapy initiation delay and other comorbidities except diabetes mellitus.

The limitations of our study consist of the lack of information regarding patients with impaired fasting glucose (IFG), which would have led us to a better understanding ofglucose metabolism disorder in a viral infectious context.

## 5. Conclusions

From our observations, patients who received Tocilizumab very early, in the first moments after sudden oxygen desaturation, had a higher chance of recovery with a favourable evolution, with a significantly higher percentage in diabetic patients compared to non-diabetics. The episode of desaturation was preceded by a clear increase in IL-6 values, a finding that could help anticipate the “cytokine storm” and the early initiation of appropriate therapy considering that the risk of developing severe forms of disease with acute respiratory failure was 1.5 times higher in diabetic patients.

Diabetic patients are more exposed to inflammatory complications during COVID-19 than those who do not have DM.

The unfavourable outcome of patients with diabetes mellitus could be due also to glycaemic disorders induced by corticoid usage, even if corticotherapy is claimed to be efficient in hyperinflammatory responses generated by SARS-CoV-2.

We did not find any notable differences in the form of pneumonia in patients with diabetes compared to non-diabetic patients, but the mortality rate was three times higher in patients with diabetes and COVID-19 compared to non-diabetics.

The mortality rate in ICU was over 60%, while in those with acute respiratory failure who received treatment with Tocilizumab, it was 27% only. The administration of IL-6 antagonists significantly reduced the need for ICU admission.

## Figures and Tables

**Table 1:** Prevalence of deaths according to studied months.

Month	No.	%
March	10	4
April	24	9.6
May	25	10.7
June	30	11.9
July	24	9.6
August	33	13
September	49	19.6
October	55	22.6

**Table 2:** Distribution of deaths by age group.

COVID-19 Deceased Patients (*n* = 250)	No	%
<40 years	2	0.8
40–49 years	18	7.2
50–59 years	36	14.4
60–69 years	58	23.2
70–79 years	72	28.8
80–89 years	55	22.0
90+ years	9	3.6

**Table 3:** Comparative distribution of deaths by age group.

COVID-19 Deceased Patients (*n* = 250)	Females	Males	Entire Group	*p* Values for Chi^2^ Test
Age < 65	16	60	76	0.037
Age > 65	59	115	174	

**Table 4:** Comorbidities of diabetic/nondiabetic patients with COVID-19.

COVID-19 Patients with Type 2 DM + (*n* = 634)	No (%)	COVID-19 Patients Non-DM (No = 3351)	No (%)	*p* Values for Chi^2^ Test
High Arterial Blood Pressure	197 (31.1%)	High Arterial Blood Pressure	825 (24.6%)	0.001
Obesity	155 (24.5%)	Obesity	626 (18.6%)	0.001
Chronic Kidney Disease	90 (14.2%)	Chronic Kidney Disease	611 (18.2%)	0.014
Neoplasia	79 (12.5%)	Neoplasia	590 (17.6%)	0.050
Autoimmune diseases	28 (4.4%)	Autoimmune diseases	117 (3.5%)	0.254
Liver Cirrhosis	20 (3.2%)	Liver Cirrhosis	99 (3.0%)	0.786

**Table 5:** Demographic characteristics in deceased patients with/without diabetes mellitus.

COVID-19 Deceased Patients (*n* = 250)	Age < 65	Age > 65	Males (no/%)	Females (no/%)	ICU Admission
with DM (*n* = 91)	25 (28.1%)	66 (71.9%)	53 (57.8%)	38 (42.2%)	66 (71.9%)
without DM (*n* = 159)	53 (33.6%)	106 (66.4%)	96 (60.2%)	63 (39.8%)	113 (70.8%)

**Table 6:** Clinical evolution characteristics of diabetic vs. non-diabetic COVID-19 patients.

COVID-19 Patients	Total Number of Patients (*n* = 3985)	with DM (*n* = 634)	without DM (*n* = 3351)	Chi^2^ Test			
				χ^2^	*p*	RR	IC95%
Severe form(respiratory failure)	870	193 (30.4%)	677 (20.2%)	32.15	0.001	1.51	1.32–1.73
chest X-ray picture suggestive for bronchopneumonia	144	20 (3.2%)	124 (3.2%)	0.001	0.938	1.00	0.62–1.58
chest X-ray picture suggestive for interstitial pneumonia	383	54 (8.5%)	329 (8.4%)	0.005	0.993	1.01	0.76–1.34
patients treated with Tocilizumab	349	103 (16.3%)	246 (7.3%)	52.89	0.001	2.21	1.97–2.74
deaths number	250	91 (36.4%)	159 (53.4%)	82.08	0.001	3.03	2.37–3.86

**Table 7:** Interleukin-6 values in COVID-19 patients.

Covid-19 Patients	IL-6 Normal Values (<3.8 pg/mL)	Il-6 < 50 pg/mL	Il-6 > 50 pg/mL
With DM (*n* = 634)	202 (31.9%)	174 (27.4%)	258 (40.7%)
Without DM (*n* = 3351)	2056 (61.4 %)	375 (11.2%)	920 (27.5%)

**Table 8:** Evolution of SARS-CoV-2 patients treated with Tocilizumab.

COVID-19 Patients	Favourable Outcome	Deaths	*p* Values for Chi^2^ Test
Patients with DM treated with Tocilizumab (*n* = 103)	79 (76.7%)	24 (23.3%)	0.001
Nondiabetic patients treated with Tocilizumab (*n* = 246)	87 (35.3)%	159 (64.6%)	

## Data Availability

The data supporting the conclusion of this manuscript will be made available by the authors without undue reservation to any qualified researcher.
